# Undertaking Research in Other Countries: National Ethico-Legal Barometers and International Ethical Consensus Statements

**DOI:** 10.1371/journal.pmed.0040010

**Published:** 2007-02-27

**Authors:** Loane Skene

## Abstract

Is it ethical for scientists to conduct or to benefit from research in another country if that research would be unlawful, or not generally accepted, in their own country?

There are many reasons for scientists to undertake research with colleagues in other countries. They may benefit from sharing knowledge and experience with colleagues from different backgrounds. They may obtain funding that is directed to transnational projects. They may gain access to more diverse facilities and participants in research. They may acquire kudos, academic advancement, or commercial benefits from an enhanced international reputation. And they may also be able to undertake activities in another country that would not be permitted in their own country, due to legal or ethical constraints.

In most cases, it will be lawful in their own country to undertake such activities overseas. It is rare for countries to have laws directly preventing their nationals doing research overseas that would not be permitted at home, or even bringing back the products of such research, unless they pose a safety risk, such as importing genetically manipulated organisms created overseas. However, some people have asked whether it is ethical for scientists to do research overseas in order to benefit from a favourable regulatory scheme and whether there should be laws to prevent such research.

This paper argues that, in the great majority of cases, there are no ethical reasons to prevent scientists from doing research abroad or using the research results at home, even if the research does not comply with local laws. To illustrate my argument, the paper focuses particularly on human embryonic stem cell research and a project in which international and multi-disciplinary experts agreed on a consensus statement setting out principles for transnational stem cell research [1]. The group of experts, known as the Hinxton Group, consisted of 60 scientists, doctors, philosophers, lawyers, scientific journal editors, federal regulators, and others from 14 countries, who attended a meeting sponsored by the Wellcome Trust at Hinxton near Cambridge, United Kingdom in February 2006 (the project is described in [2]). The group developed and unanimously endorsed a set of international principles (summarised in Box 1), even though the law and the ethical procedures in their respective countries often differed.

**Box 1. Some Ethical Principles Agreed Upon by the Hinxton Group**

The consensus statement published by the Hinxton Group said that stem cell research “holds out immense promise for good” but acknowledged “the reality of cultural diversity and moral disagreement” about some aspects of it. It said that “Law makers should be circumspect when regulating science”; laws should be flexible to “accommodate rapid scientific advance”; and laws should not be extraterritorial. The statement set out the following ethical responsibilities of scientists, journal editors, and scientific academies and organisations (which I have paraphrased).

- *Scientists* should conduct research only if it is scientifically and ethically defensible and undertaken according to ethically accepted norms, especially protecting the well-being, liberty, and rights of cell and tissue donors and research participants. Scientists should obtain valid informed consent from research participants and address conflicts of interest. If requested by editors, they should provide protocols for ethics review, consent forms, and information statements. They should submit any stem cell lines they derive to national or international depositaries and make cell lines and data publicly available.
- *Journal editors* should promote high standards for scientific peer review. They should require authors to state whether their research complies with local laws and policies, including ethical oversight. They should encourage authors to include in manuscripts explicit descriptions of their roles in the published research and to submit data verifying the source of cells, the authenticity of the cell line(s), and how the scientists have complied with good cell culture practice.
- *Academies of science and professional organisations* in consultation with the public should continue to develop guidelines for the ethical conduct of stem cell research and clinical trials, including the “challenges of international collaboration”. A public database should be established of “statements of ethical conduct and guidance, research protocols, consent forms, [and] information provided to potential human subjects and tissue donors…”

My central argument is that when research done overseas falls above a certain level on a country's “ethico-legal barometer” (see Figure 1), it should be assumed that there is not such a high level of ethical objection within the country as to make its ethical standards significantly different from those reflected in the international consensus statement. However, when the proposed research falls in the red zone of the barometer (very widely condemned; laws with extraterritorial application), compliance with the consensus statement would not reassure people in the home country. Such “red zone” research done overseas may be regarded as unethical and should be prohibited. If scientists in the home country seem inclined to do “red zone” research in other countries, the home country can enact extraterritorial laws to prevent them doing so.

**Figure:**
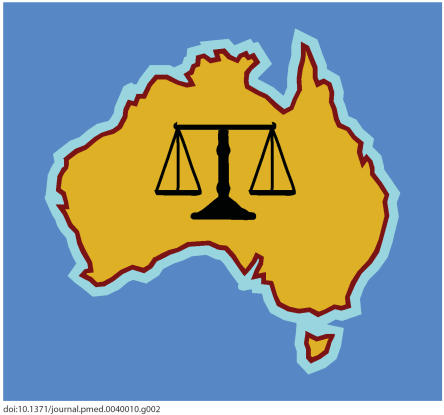
Each country's laws generally apply only within its boundaries; laws with extraterritorial effect are enacted only when an activity is widely condemned (e.g., paedophilia)

## Issues in Transnational Research: A Scenario

Assume that an Australian scientist wants to obtain and study stem cells from a human embryo created by somatic cell nuclear transfer (SCNT), so that the cells are immunologically compatible with those of the person whose somatic cells are used to create the embryo. In Australia, it is unlawful to create an embryo by SCNT and derive stem cells from it, but in the UK, those practices are lawful.

Is there any legal or ethical reason why the Australian scientist should not be permitted to conduct the research in the UK, or to use the stem cells or research data obtained in the UK when returning to Australia? Could the researcher, his or her employer, or an Australian or UK agency that funds the research, face legal liability for a possible breach of Australian law? Can ethical concerns in Australia about the proposed research be placated by knowing that the research has undergone scientific and ethical scrutiny in the UK?

## The Law

The law is perhaps clearer than the ethical aspects of such cases. In the scenario above, the Australian scientist (and others associated with the project) will be acting lawfully if the research undertaken in the UK is lawful in that country, even if it is not lawful in Australia. It is a fundamental exercise of the notion of sovereignty which underpins international law for states (such as Australia and the UK) to determine and enforce applicable law within their own physical territory. Foreign nationals visiting or working in another country are primarily subject to the laws of that host country. Governments consistently warn their nationals intending to travel abroad of the need to respect the laws of the countries they visit or suffer the consequences under that local law.

There are some exceptions to this general principle. The Australian government could restrict the use within Australia of research data or material obtained in another country, in the same way as it limits the importation of biological material under its quarantine and customs legislation. This means that the Australian scientist could not benefit from using the stem cells in further research in Australia but would not be committing any offence against Australian law by doing the research in the UK. Similarly, an employer or funding body would not be an accessory to criminal activity, as no crime has been committed. There would only be a crime if a law was passed in Australia preventing scientists from undertaking the research in another country.

International law does recognise that governments can exercise national jurisdiction “extraterritorially”—that is, beyond the physical territory of their country. In Australia, this must be specifically stated in the relevant legislation; silence means that it applies only within Australia. National jurisdiction can be extended on the basis of a person's nationality rather than the physical territory of the country where they engaged in their activity. Most countries (including the UK and Australia) have legislation extending the application of their criminal law to acts of their military personnel engaged in operational deployment abroad. Other examples of laws imposing criminal liability for nationals' acts overseas include violations of the Chemical Weapons Convention and “sex tourism” (paedophilia).

Thus, countries can extend their territorial jurisdiction, either by penalising acts of their nationals committed abroad, or by preventing their nationals from using the products or results of work undertaken overseas when they come back. In practice, however, such laws are rare and they will only be made when other countries have very different domestic laws and the activity is one that they really condemn (such as the development of chemical weapons or paedophilia).

Although the laws on embryonic stem cell research vary between countries, no country to date has tried to enact legislation to directly extend its territorial jurisdiction in this area. The Council of Europe's Convention on Human Rights and Biomedicine prohibits the creation of embryos for research purposes [3]. However, a convention operates as law only if it is ratified by a particular country and is made a national law. It will have extraterritorial effect only if specified in the legislation. No country has done that. At most, countries have extended their extraterritorial “reach” by preventing the importation of certain bodily material, or by attaching conditions to funding of research overseas. For example, all recipients of United States government funds for research are required to follow applicable US regulations on research involving human participants, even if the research is done in other countries. The US restrictions apply only to government-funded or government-regulated research, not to research funded by private agencies. Also, problems may arise where there are multiple funders or the US regulations conflict with local laws. In such cases, the effectiveness of funding restrictions is varied in extraterritorial regulation. If the research is supported solely by federal funding, the US regulations must be obeyed; if scientists receive non-federal funding, they can do research that would otherwise be prohibited.

Presumably the reason that countries have not directly enacted extraterritorial laws on stem cell research is that there is not sufficient condemnation in the researcher's home community of the stem cell research that is permitted in other countries (see Figure 1). Indeed, the same is true in other areas of science and medical practice. Australia, for example, has not legislated to prevent its nationals going to the US to undergo commercial surrogacy or to use commercially obtained eggs for infertility treatment, despite these practices being prohibited in Australia. There is no legislation preventing the use of research results from trials in developing countries, even if they fall short of Australian requirements. A foreign research project involving victims of capital punishment would not be allowed in Australia (where capital punishment is not permitted), but Australia is unlikely to enact legislation preventing that research in other countries (where it may be lawful) or the use of results from such research in Australia. It is only where there is a public safety risk (as with the importation of genetically modified organisms) that laws have been enacted to prevent importation of a product of research, and they do not extend to doing the research in another country.

Indeed, it is only with widely condemned practices, such as paedophilia and the making of chemical weapons, that countries have legislated to prevent their nationals doing those things overseas. There is no extraterritorial legislation on human embryo research, paying volunteers in clinical trials, buying eggs for fertility treatment, or engaging a paid surrogate mother. Views might differ on the acceptability of all these practices and even if they are not permitted in Australia, the community has not called for legislation to prevent Australian nationals doing them abroad. It is only in extreme cases that legislation might be demanded to prevent such practices as, for example, research on victims of torture. However, even in such a case, a country will propose extraterritorial legislation only if its nationals appear likely to engage in such practices.

## Ethical Principles

Whatever the law may be, however, the issues are more problematic if one takes an ethical, rather than a legal, view. Even if it is lawful for an Australian scientist to do research in the UK that is not allowed in Australia, is it ethical for Australian scientists to do that in order to avoid the ethical constraints in their own country? To what extent can one adopt a relativist approach: *autres pays, autres mores* (“other countries, other customs”)?

In considering this question, it is useful to imagine a continuum of conduct—a national ethico-legal barometer (Figure 1). Conduct in the red zone is prohibited by both national and extraterritorial laws; in the orange, prohibited only by national laws; in the yellow, permitted subject to national laws and ethical oversight; and in the green, permitted subject to ethical oversight. The two relevant areas are the red and the orange; in the others, there is no legal prohibition.

### The red zone.

For red zone activities, a relativist approach seems unacceptable. The conduct in the other country would not only be unlawful in Australia but would be widely condemned on ethical grounds, even if it were lawful in the country in which it occurs. However, more interesting issues arise if one adds some activities from the bottom of the orange zone.

Imagine that an Australian scientist wants to undertake reproductive cloning in a country where that is not forbidden by law and wants to develop the technique back in Australia. Such research would be unlawful in Australia and almost universally regarded as ethically unacceptable, particularly because of the unknown risks to any child born from such a procedure. Similarly, research that imposes severe suffering on animals, especially for a cosmetic rather than scientific purpose, would be widely condemned as well as unlawful in Australia, as would research that involves killing animals from rare and endangered species.

Even if these types of research were allowed in another country and arguments could be advanced to support them, Australians might reject a relativist approach and not allow the results to be used in Australia (though if there were a real benefit from the research, the initial repugnance might be overcome, which involves different moral issues). If activities were widely regarded as morally or ethically wrong, then no requirement of scientific rigour or ethical oversight in the other country would make them acceptable in Australia. Thus, although activities such as reproductive cloning are not currently the subject of extraterritorial laws, the law could be changed. If Australian scientists seemed inclined to undertake those activities abroad, the country's ethico-legal barometer might rise, with extraterritorial legal restrictions being imposed on Australian nationals working abroad.

### The orange zone.

For conduct that falls within the orange zone, one might adopt a relativist approach. Although unlawful in Australia, such activities are not so much condemned as to require extraterritorial effect and may in fact be condoned by many Australians, even if a majority disapproves of them. Creating a human embryo by SCNT, for example, is unlawful in Australia, but many people support the English position where this is allowed and supporters can give detailed ethical reasons for their views.

Supporters of SCNT argue that SCNT embryos are not true embryos because they are not formed from male and female gametes; they are not intended for use in a pregnancy; and they are not viable without extended scientific support. Alternatively, even if SCNT embryos are not regarded as being essentially different from “sperm–egg embryos”, it is ethically justifiable to use both kinds of embryos in research for a limited period subject to strict regulatory controls and ethical oversight because it may yield important scientific knowledge and perhaps lead to cures for serious genetic conditions. Indeed, it is ethically inconsistent to allow “surplus” embryos from fertility programs to be used in research (which is lawful in Australia as well as in the UK), but not specially created and non-viable SCNT embryos.

In the orange zone, where there are no laws preventing scientists undertaking research in other countries and ethical views are divided, objectors may be reassured by the requirements of scientific and ethical scrutiny of the research in the other country. This was the starting point for the Hinxton Group who were able to agree on a consensus statement, despite its members coming from many different countries, with different laws and ethical principles [1]. The principles in the statement are based on generally agreed-upon ethical concepts, many of which are not expressly set out in the statement but incorporated by reference to “ethically acceptable norms”.

These principles include observance of the practices of scientific integrity; not exposing participants, including cell and tissue donors, to unreasonable risks; fully informing research participants before they consent; and openness to ethical review. The principles are worded in general terms, like the international statements [4,5] and ethical principles on which they are based, and, like the international statements, they are silent on the creation of embryos for research purposes. (Both the Declaration of Helsinki [4] and the Council for International Organizations of Medical Sciences' *International Ethical Guidelines for Biomedical Research Involving Human Subjects* [5] do not discuss embryo research. The latter guidelines note that “An attempt to craft a guideline on the topic proved unfeasible. At issue was the moral status of embryos and fetuses and the degree to which risks to the life or well-being of these entities are ethically permissible”.)

The Hinxton principles do not even suggest that human embryos have a special moral significance. They do not require, for example, that the use of embryos in research and the number used should be justified. The only relevant statement that could perhaps apply to embryos is that “any risk of harm should be commensurate with expected overall benefit”, and that might refer to the need to protect women from exploitation as egg donors, or other research participants from potential harms.

## Conclusion

How reassuring are broad ethical principles in international consensus statements, such as the one produced by the Hinxton Group, for people who have deeply held ethical objections to the type of research that is conducted in other countries? As noted, the issue of greatest contention, the creation of embryos for research, is not addressed in the Hinxton statement. Also, objectors are asked to take on faith that research will be scientifically and ethically monitored under another country's regime, but this monitoring cannot be checked or enforced in another country as it can in the home country.

In Australia, for example, human embryo research is monitored by a federal licensing committee and government inspectors with broad powers to enforce the legislation. If the scientific integrity of a project is questioned, or there is any doubt about whether participants entered the project freely after proper information and consent, then those matters can be investigated by calling for the records of the project, including the plain language statement and the consent form. Similarly, it would be possible for the government authorities responsible for testing and approving new drugs and procedures to examine the records to check the accuracy of scientific data. That is not the case when research is done in another country. Even if there is provision in that country for external examination of records associated with a research trial, the procedure cannot be instigated or enforced by Australian authorities. Also, the review that may be needed for quality assurance of imported stem cell lines and other biological substances depends largely on the cooperation of the collaborators in other countries.

On the other hand, the principles and procedures approved by the Hinxton Group do suggest that countries can agree to adopt a similar approach to particular types of research, at least in relation to scientific and ethical oversight being the best form of regulation rather than national laws. The real issue is how helpful such principles will be when there are wider and more deeply held differences of opinion about particular types of research. With human embryo research, views may vary about what is permissible or people may agree to differ on the source of embryonic stem cells for research. Issues may then be resolved by general, fairly non-contentious principles for scientific and ethical oversight, such as those in the Hinxton statement. For research that is even more contentious, where orange zone activities may be made red ones, one cannot imagine that a consensus approach would be possible.

## 

**Figure 1:**
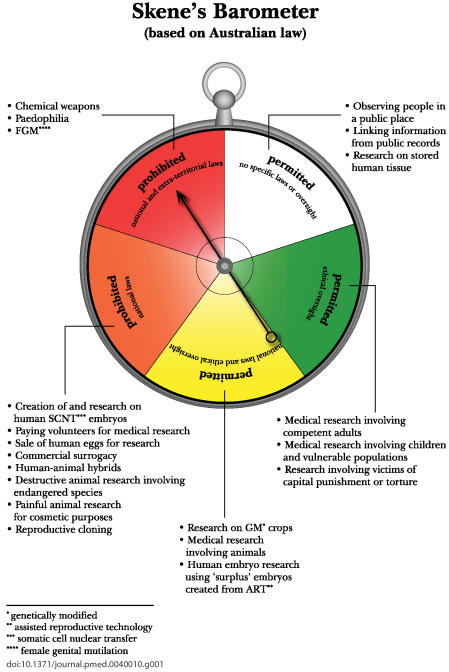
The Ethico-Legal Barometer: Australia (illustration: Anthony Flores and Loane Skene)

## Abbreviations

SCNT: somatic cell nuclear transfer
